# Revisiting the Role of Interleukin-1 Pathway in Osteoarthritis: Interleukin-1α and -1β, and NLRP3 Inflammasome Are Not Involved in the Pathological Features of the Murine Menisectomy Model of Osteoarthritis

**DOI:** 10.3389/fphar.2017.00282

**Published:** 2017-06-13

**Authors:** Sonia Nasi, Hang-Korng Ea, Alexander So, Nathalie Busso

**Affiliations:** ^1^Département de l'appareil Locomoteur, Service of Rheumatology, Centre Hospitalier Universitaire Vaudois and University of LausanneLausanne, Switzerland; ^2^Institut National de la Santé et de la Recherche Médicale, UMR-1132, Hospital LariboisièreParis, France; ^3^Departement de Rhumatologie, Université Paris Diderot (UFR de Médecine)Paris, France

**Keywords:** NLRP3 inflammasome, interleukin-1β, cartilage, knock-out mice, animal model of OA

## Abstract

**Background:** Innate immune response components such as toll-like receptors (TLRs) and NLRP3-inflammasome act in concert to increase IL-1α/β secretion by synovial macrophages. Previous results suggest that IL-1α/β could be an important mediator involved in the pathogenesis of osteoarthritis (OA).

**Objectives:** The aim of our study was to evaluate the role of NLRP3, IL-1β, and IL-1α in the menisectomy (MNX) model of murine OA.

**Methods:** Murine chondrocytes (CHs) and bone marrow-derived machrophages (BMDM) were stimulated with hydroxyapatite (HA) crystals, a form of calcium-containing crystal found in human OA, and IL-1β and IL-6 secretion assayed by ELISA.Conversely, the ability of IL-1β and IL-6 to induce CHs calcification was assessed *in vitro* by Alizarin red staining. Knees from 8 to 10 weeks old C57Bl/6J wild-type (WT) (*n* = 7), NLRP3^−/−^ (*n* = 9), IL-1α^−/−^ (*n* = 5), and IL-1β^−/−^ (*n* = 5) mice were menisectomized, using the sham-operated contralateral knee as control. 8 weeks later, knee cartilage degradation and synovial inflammation were evaluated by histology. In addition, apoptotic chondrocytes, metalloproteases activity, and collagen-type 2 expression were evaluated in all mice. Joint calcification and subchondral bone parameters were quantified by CT-scan in WT and IL-1β^−/−^ menisectomized knees.

**Results:** *In vitro*, HA crystals induced significant increased IL-6 secretion by CHs, while IL-1β remained undetectable.Conversely, both IL-6 and IL-1β were able to increase chondrocytes mineralization. *In vivo*, operated knees exhibited OA features compared to sham-operated knees as evidenced by increased cartilage degradation and synovial inflammation. In menisectomized KO mice, severity and extent of cartilage lesions were similar (IL-1α^−/−^ mice) or exacerbated (IL-1β^−/−^ and NLRP3^−/−^ mice) compared to that of menisectomized WT mice. Metalloproteases activity, collagen-type 2 expression, chondrocytes apoptosis, and synovial inflammation were similar between KO and WT mice menisectomized knees. Moreover, the extent of joint calcification in osteoarthritic knees was comparable between IL-1β^−/−^ and WT mice.

**Conclusions:** MNX knees recapitulated features of OA, i.e, cartilage destruction, synovial inflammation, cell death, and joint calcification. Deficiency of IL-1α did not impact on the severity of these features, whereas deficiency of IL-1β or of NLRP3 led to increased cartilage erosion. Our results suggest that IL-1α and IL-1β are not key mediators in this murine OA model and may explain the inefficiency of IL-1 targeted therapies in OA.

## Introduction

Osteoarthritis (OA) is a progressive disease of the joint tissues, characterized by cartilage degradation (Goldring and Goldring, 2007; Loeser et al., 2012), mild synovial inflammation (Scanzello and Goldring, 2012), subchondral bone sclerosis, osteophyte formation, and calcium crystal deposition (calcification) on cartilage (McCarthy and Cheung, 2009). Several OA risk factors have been identified including joint trauma, aging, sex, genetics, obesity (Abramson and Attur, 2009), and basic calcium phosphate (BCP) crystals in joints (McCarthy and Cheung, 2009). BCP crystals include octacalcium phosphate (OCP), hydroxyapatite (HA), and carbonated-apatite (CA) crystals (Ea and Liote, 2009), the latter being the most abundant (Gibilisco et al., 1985). Although OA is the most common form of joint disease and a leading cause of disability in the elderly (Goldring, 2006), no drug exists to slow the progression, or reverse the OA disease process (Iqbal and Fleischmann, 2007).

There is evidence that articular tissues in OA produce proinflammatory cytokines such as IL-1 and IL-6. IL-6 is synthesized and secreted in an active form, which binds first to its receptor (IL-6R) and then to the signaling gp130 molecule triggering STAT and ERK pathways. In contrast, both IL-1α and IL-1β exist as an intracellular proform of about 31 kDa, which can be cleaved to a mature form of 17 kDa. In particular, a first signal (such as TLR1/2 agonist PAM3Cys or TLR4 agonist LPS) is needed to trigger an NF-kB–dependent production of pro–IL-1β. A second signal (ATP, BCP crystals, and others) then leads to the assembly and oligomerization of the NLRP3 inflammasome composed of the NLRP3 sensor, the adaptor protein ASC (Apoptosis-associated speck-like protein containing a CARD) and procaspase-1 which is activated in caspase-1 (Broz and Dixit, 2016). Caspase-1 and NLRP3 inflammasome facilitate or are needed for proIL-1β proteolytic processing and release (Gross et al., 2012), but pro-IL-1α is mainly processed by calpain and other proteases (Di Paolo and Shayakhmetov, 2016). Cellular activation due to signaling through the IL1-R1 occurs when either IL-1α and/or IL-1β (which are equally potent cytokines, collectively known as IL-1) bind to the widely expressed IL-1R type 1 (IL-1R1). Binding induces the formation of a high-affinity complex with the IL-1R accessory protein (IL-1RAcP) and the recruitment of the intracellular adaptor protein myeloid differentiation factor 88 (MyD88) and of the IL-1R-associated kinase 1 (IRAK), which are the proximal mediators of IL-1 signaling. Uncontrolled activation of IL-1R1 is prevented by two distinct mechanisms. One is mediated by IL-1Ra that competes with IL-1 for binding to IL-1R1, and blocks intracellular signaling and cell activation. The other is via the type 2 IL-1 decoy receptor (IL-1R2) that acts as a trap for IL-1 but, in contrast to IL-1R1, lacks a cytoplasmic domain and is unable to induce signaling (Re et al., 1996).

*In vitro*, in joints cells (such as fibroblasts, macrophages, chondrocytes, osteoblasts), IL-6 and IL-1 are responsible for the loss of cell metabolic homeostasis by 1-promoting autocrine induction of cytokines or production of other inflammatory compounds or chemokines, 2-inducing matrix-degrading enzymes such as MMP-1,-3,-9,-13, and -14 and ADAMTS4,5, and 9 (Murphy and Nagase, 2008; Hashizume and Mihara, 2010; Wojdasiewicz et al., 2014) and, 3-inhibiting the expression of a number of genes, such as collagen type 2 gene (Col2a1) (Goldring et al., 1988; Poree et al., 2008), and proteoglycan (van Beuningen et al., 1991; Sui et al., 2009), normally associated to healthy chondrocytes. Additionally, both IL-1 and IL-6 have pro-mineralizing activity in chondrocytes (Johnson et al., 2001; Nasi et al., 2016a). Finally IL-1β and IL-6 exert their catabolic effect also in bone, by inducing differentiation of mononuclear precursors in osteoclasts (Jandinski, 1988; Nakamura and Jimi, 2006; Kim et al., 2009) and by stimulating bone resorption activity by osteoclasts via the receptor-activator of NF-kB ligand (RANKL) (Jules et al., 2012). Altogether, these *in vitro* effects of IL-1 and IL-6 strongly suggest that these cytokines should have a deleterious role in OA progression *in vivo*, inducing synovitis, favoring cartilage degradation by both catabolic and anabolic effects, and promoting cartilage calcification.

In synovial fluids of either human or experimental models of OA, both cytokines were found to be increased and correlated with radiographic knee OA (Livshits et al., 2009; McNulty et al., 2013). However, IL-1 was not significantly overexpressed in moderate OA compared to mild OA (McNulty et al., 2013). In experimental murine OA, IL-6 neutralization, its genetic deficiency or inhibition of its signaling molecule Stat3 with a non-peptidic small molecule prevented cartilage damage (Ryu et al., 2011; Latourte et al., 2016). In addition, in menisectomized mice, increasing deposits of BCP-crystals were observed around the joint and correlated with cartilage degradation and IL-6 expression (Nasi et al., 2016a). While most of the studies clearly report a deleterious role of IL-1 in *in vitro* models of OA, many discrepancies exist about IL-1 effects and IL-1 blockade in experimental OA (see summary in **Table 2**). In particular, using genetically deficient mice, Glasson et al. reported that IL1β^−/−^ mice were protected in a surgical induced instability model of OA (Glasson, 2007), whereas Clements found in a similar model that cartilage damage was exacerbated in Caspase1^−/−^ and IL1β^−/−^ mice (Clements et al., 2003). More recently, in the collagenase-induced model of OA (CiOA), IL-1αβ^−/−^ mice were not protected against synovial inflammation and cartilage destruction if compared to WT mice. Moreover, intra-peritoneal injection of IL-1Ra in WT osteoarthritic mice did not ameliorate OA features (van Dalen et al., 2016).

Due to the confusing published results concerning the role of IL-1 in experimental models of OA, we have further reexplored its role and the role of NLRP3 inflammasome in IL-1 activation in the context of surgically induced murine OA.

## Methods

### Mice and induction of experimental osteoarthritis

IL-1α^−/−^, IL-1β^−/−^, and NLRP3^−/−^ female mice, all in the C57Bl/6J background (obtained by Prof Fabio Martinon, Epalinges, Switzerland), were compared with WT littermates. Body weight, fertility and viability were similar among different genotypes. Mice between 8 and 10 weeks were anesthetized and knee joint instability was induced surgically by medial partial meniscectomy of the right knee, as previously described (Nasi et al., 2014). The contralateral knee joint was sham-operated and used as internal control. The animals were allowed unrestricted activity, food and water *ad libitum* in a pathogen-free housing facility. This study was carried out in accordance with the guidelines set by the “Service de la consommation et des affaires vètèrinaires du Canton de Vaud.” The protocol was approved by the Federal Veterinary Office and the work complied with the Directive 2010/63/EU.

### Histology of total knee joints

Total knee joint of mice were fixed, decalcified and embedded in paraffin, and sagittal sections were cut from the whole medial compartment of the joint (three sections/mouse) as previously described (Nasi et al., 2014). Sections were then stained with Safranin-O-fast green to examine the OA-like cartilage and bone changes according to the scoring method recommended by OARSI (Glasson et al., 2010). Finally, synovial inflammation was scored using the following scale: 0=no inflammation; 1=mild inflammation; 2=moderate inflammation; 3 = major inflammation. Synovial histological changes included synovial hypertrophy and hyperplasia and an increased number of lining cells, accompanied sometimes by infiltration of the sublining tissue. Histological scorings were assessed by two observers who were blinded with regard to the mice genotypes.

### MicroCT-scan

MicroCT-scans analysis were performed using a SkyScan 1076® X-ray μCT scanning system (SkyScan, Belgium) and the following parameters: 18 μm resolution, 60 kV, 167 μA, 0.4° rotation step over 360°, 0.5 mm Aluminum filter, 1180 ms exposure time. *Ex vivo* samples acquisition was made using formol fixed knees. Images were reconstructed using NRecon Version 1.6.6.0 (Skyscan, Belgium) considering the following parameters: gray-values = 0.0000–0.105867, Ring Artifact Reduction = 3, Beam Hardening Correction = 40%. In the menisectomized knees, quantitative analyses of crystal content (μg), and quantitative analysis of tibial subchondral bone parameters (bone mineral density (BMD g/cm^3^), trabecular thickness (Tb.Th), trabecular number (Tb.N), and trabecular spacing (Tb.Sp) were performed using CTAnalyzer Version 1.10 (SkyScan, Belgium) for different Volumes Of Interest (VOIs).

### Immunohistochemical detection of VDIPEN, type II collagen and apoptosis

MMP-induced neoepitope VDIPEN staining was performed with affinity-purified anti-VDIPEN IgG and type II collagen synthesis was evaluated using an anti-collagen type II, biotinylated monoclonal antibody (MD Bioproduct, 1041007B) (Nasi et al., 2014). Apoptotic chondrocytes were detected in paraffin sections using the Apoptag kit (ApopTag plus Peroxidase *In situ*, Millipore) as previously described (Nasi et al., 2014).

### Calcium phosphate crystals

Hydroxyapatite (HA) crystals were synthesized as previously published (Prudhommeaux et al., 1996). HA crystals were sterilized by gamma-radiation and assessed as pyrogen-free. Prior to experimentation, crystals were resuspended in sterile PBS and sonicated for 5 min.

### Bone marrow derived macrophage (BMDM) preparation

Bone marrow cells were isolated from the tibia and femur of C57BL/6 mice. For their differentiation into BMDM, the extracted cells were incubated for 7 days in Petri dishes with 30% L929 conditioned media (source of M-CSF) and 20% FBS in Dulbecco's Modified Eagle Media (DMEM). The resulting BMDM were detached using cold PBS, plated in complete DMEM medium (Gibco), [10% FBS and 1% Penicillin Streptomycin (Sigma)] or incomplete DMEM (1% Penicillin Streptomycin only) and primed or not with 100 ng/ml PAM3Cys overnight. The following day, crystal stimulation was performed in incomplete DMEM.

### Joint chondrocyte (CHs) preparation

Chondrocytes were isolated from new-born C57Bl/6J mice as described previously, with slight modifications (Gosset et al., 2008). Briefly, the joint cartilage (articular and epiphyseal) was harvested from the knee and hip joints of mice aged between 4 and 6 days. The cartilage was degraded by a three step digestion process by using decreasing concentrations of Liberase (Roche). The day after, the digested tissue was passed through a 70 μm filter (BD biosciences) to obtain immature chondrocytes. The cells were plated into a culture plate at high density (3.5 × 10^4^ cells/cm^2^) and amplified for 7 days in complete DMEM (10% FBS, 1% Penicillin Streptomycin). Prior to crystal stimulation experiments, cells were detached using Trypsin-EDTA (Amimed). The resulting chondrocytes were plated in complete DMEM medium (Gibco), [10% FBS and 1% Penicillin Streptomycin (Sigma)] or incomplete DMEM (1% Penicillin Streptomycin only) and primed or not with 100 ng/ml PAM3Cys overnight. The following day, crystal stimulation was performed in incomplete DMEM. For chondrocyte mineralization analysis, cells were cultured for 7 days in complete BJGb medium (Gibco) (10% FBS, 50 μg/ml ascorbic acid, 20 mM β-glycerol phosphate), stimulated or not with 10 ng/ml of IL-6 (Gibco PMC0064) or with 1 ng/ml IL-1β (Gibco PMC0814). Medium was changed for the last 4 days.

### Calcium phosphate crystal stimulation

Cells were primed overnight with100 ng/ml Pam3Cys, where indicated, and stimulated with 500 μg/ml HA crystals. Supernatants were collected for cytokine ELISAs, and cells placed in TRIZOL for RT-PCR analysis.

### Crystal detection from chondrocyte cultures

Articular chondrocytes cultured for 7 days were washed in PBS and crystal deposition analyzed as previously described (Gregory et al., 2004).

### PCR analysis

RNA was extracted and PCR or qRT-PCR with gene specific primers (Table 1) was performed as previously described (Ea et al., 2013).

**Table 1:** Gene specific primers for PCR and qRT-PCR analysis.

**Gene**	**Forward primer (5′ → 3′)**	**Reverse primer (5′ → 3′)**
*Asc*	CCA GTG TCC CTG CTC AGA GT	TCA TCT TGT CTT GGC TGG TG
*Casp1*	CCG TGG AGA GAA ACA AGG AG	ATG AAA AGT GAG CCC CTG AC
*Gapdh*	CTC ATG ACC ACA GTC CAT GC	CAC ATT GGG GGT AGG AAC AC
*Il-1b*	CCA CCA ACA AGT GAT ATT CTC CAT G	GTG CGG TCT TTC ATT ACA CAG
*Nlrp3*	TGC TCT TCA CTG CTA TCA AGC CCT	ACA AGC CTT TGC TCC AGA CCC TAT
*Tbp*	CTT GAA ATC ATC CCT GCG AG	CGC TTT CAT TAA ATT CTT GAT GGT C

### Cytokine quantification

Supernatants were assayed using murine IL-1β and IL-6 ELISA kit (eBioscience) following the manufacturer's protocol. Results were read at 450 nm using the Spectrax M5e (Molecular devices).

### Statistical analysis

*In vitro* experiments were performed using pools of primary cells from at least 3 different mice (either chondrocytes or bone marrow derived macrophages). Moreover, all experiments were performed with triplicates and reproduced independently at least two times. Statistical analysis was performed using the Student's *t*-test or one- or two-way ANOVA test corrected with *post-hoc* tests for multiple comparisons, where appropriate. Data was analyzed with GraphPad Prism software (GraphPad, San Diego).

## Results

### IL-1α^−/−^, IL-1β^−/−^, and NLRP3^−/−^ mice are not protected against cartilage damage and synovial inflammation induced by menisectomy

**Figure 1:**
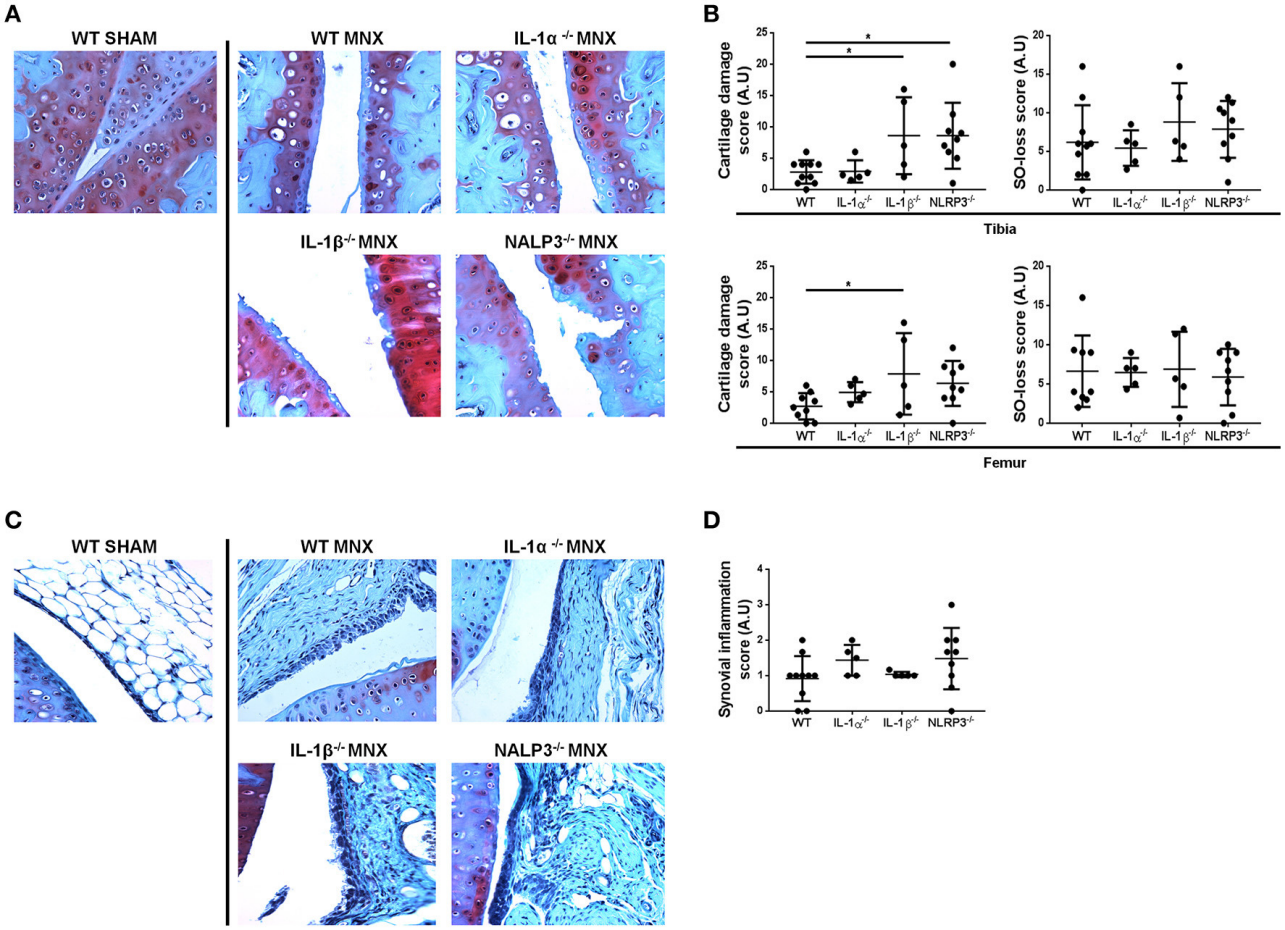
IL-1α^−/−^, IL-1β^−/−^, and NLRP3^−/−^ mice develop OA features similar to WT mice after menisectomy. **(A)** Representative histologies, stained with Safranin-O, and **(B)** respective histological scoring of sham operated (WT SHAM) and menisectomized (WT MNX, IL-1α^−/−^ MNX, IL-1β^−/−^ MNX and NLRP3^−/−^ MNX) knee joint sections, at 8 weeks after surgery. Note the similar level of cartilage destruction and loss of proteoglycans in WT and IL-1α^−/−^ menisectomized knees, and exacerbated cartilage damage in IL-1β^−/−^ and NLRP3^−/−^ MNX mice. **(C)** Representative histologies, stained with Safranin-O, and **(D)** respective synovial inflammation scoring of sham operated and menisectomized knee joint sections. Note the similar level of synovial inflammation in mice of different genotypes. Mice number: WT MNX *n* = 10, IL-1α^−/−^ MNX *n* = 5, IL-1β^−/−^ MNX *n* = 5, and NLRP3^−/−^ MNX *n* = 9. ^*^p < 0.05.

At 8 weeks after surgery, sham-operated knee joints showed intact cartilage with smooth cartilage surfaces and conserved proteoglycan (PGs) staining in both tibia and femur (Figure 1A). Chondrocytes organization was typical of that of healthy cartilage, with one or two layers of flat cells in the superficial zone and columns of round cells in the middle and deep zones. By contrast, operated knees (MNX) from WT mice exhibited cartilage damage, PGs loss and disorganized chondrocytes arrangement. In IL-1α^−/−^, IL-1β^−/−^, and NLRP3^−/−^ menisectomized mice, cartilage degradation was evidenced and similar (for IL-1α^−/−^) or more pronounced (for IL-1β^−/−^ and NLRP3^−/−^) to that of WT mice (Figure 1A). We also observed in all mice genotypes, chondrocyte morphology changes, chondrocytes loss in the superficial and intermediate cartilage layers, and chondrocytes hypertrophy in the deep zone.

We then scored the histological sections and found that the severity (grade) and the extent (stage) of cartilage degradation were similar between WT and IL-1α^−/−^, but significantly increased in IL-1β^−/−^ knees, both for the tibia and the femur cartilage (Figure 1B). Femur cartilage in NLRP3^−/−^ mice was similarly damaged but tibial cartilage was significantly increased if compared to that of WT mice (Figure 1B). Finally, levels of Safranin-O loss were similar amongst the genotypes, indicating similar levels of PG loss in cartilage of these mice. Altogether these results demonstrate that a single deficiency of IL-1α, IL-1β, or of NLRP3 does not prevent cartilage damage.

We next examined synovial inflammation in sham operated and MNX mice. We found mild synovial inflammation in all WT menisectomized mice compared to sham-operated. We also found similar synovial inflammation in IL-1α^−/−^, IL-1β^−/−^, and NLRP3^−/−^ MNX mice (Figure 1C). Synovial histological features induced by OA development included synovial hypertrophy and hyperplasia. Figure 1D shows the synovial inflammation score, which confirmed no significant differences between genotypes.

### NLRP3^−/−^, IL-1α^−/−^, and IL-1β^−/−^ mice are not protected against catabolic changes and chondrocyte apoptosis induced by menisectomy

**Figure 2:**
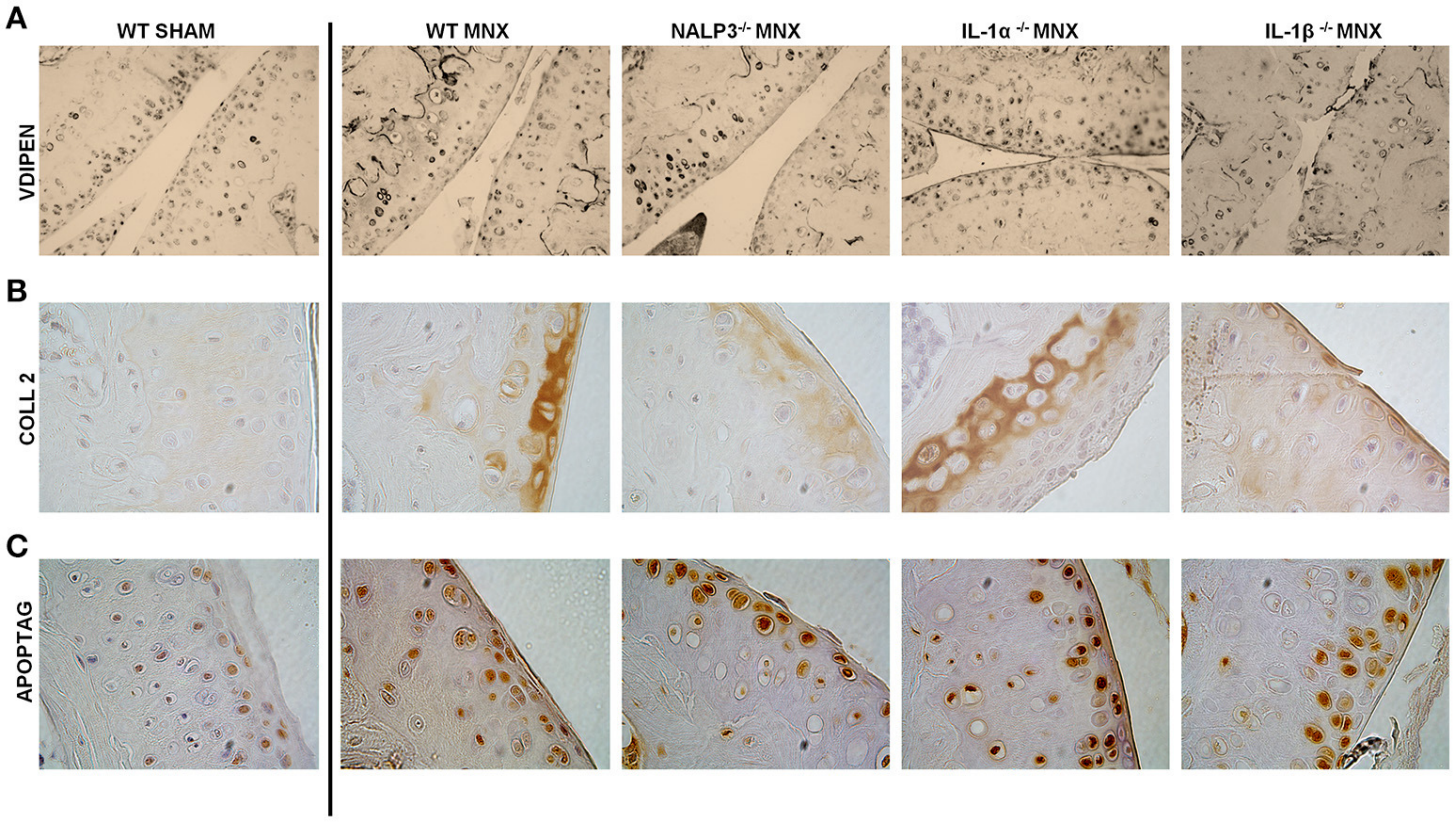
Knock-out mice are not protected against catabolic changes and chondroptosis caused by OA induction. **(A)** Representative immunohistochemical sections of knee joints, stained for MMP-mediated proteoglycan degradation (VDIPEN staining). Note markedly increased VDIPEN in MNX knees if compared to sham-operated mice and no protection in knock-out mice. **(B)** Representative immunohistochemical sections of knee joints, stained with anti collagen type 2 antibody. Note increased collagen type 2 synthesis in the superficial and deep cartilage layers of MNX mice compared to the sham operated mice and the similar expression of collagen type 2 in WT and KO menisectomized knees. **(C)** Representative immunohistochemical sections of knee joints, stained with ApopTag. Note increased apoptosis in MNX mice compared to sham operated mice and the similar level of chondrocyte aptosis in WT and KO menisectomized knees. Mice number: WT MNX *n* = 4, IL-1α^−/−^ MNX *n* = 4, IL-1β^−/−^ MNX *n* = 4, and NLRP3^−/−^ MNX *n* = 4.

Signs of cartilage catabolism, in particular of MMP-mediated aggrecan degradation, were evidences by VDIPEN staining. MMP-generated neoepitopes were markedly increased in WT MNX knees if compared to that of sham-operated mice, especially in the middle and deep zones of cartilage (Figure 2A). Moreover, both chondrocytes and pericellular matrix were associated with marked VDIPEN neoepitope immunostaining in a comparable way in WT, and NLRP3^−/−^, IL-1α^−/−^, and IL-1β^−/−^ operated mice (Figure 2A).

In addition to catabolic signs, also anabolism and in particular collagen type 2 immunostaining has been investigated. Our observations identified weak but homogeneous extracellular distribution of collagen type 2 in sham-operated mice (Figure 2B). On the contrary, osteoarthritic joints showed sites of activated collagen type 2 synthesis, in a similar way in WT and KO mice. Even if subtle differences were seen in collagen type 2 expression between the different KO mice, positivity was overall detected at a similar level and especially in the superficial damaged region as well as in the deeper layer close to the tidemark with the bone. Another prominent feature of OA is increased chondrocyte apoptosis, that we tested by TUNEL staining (Figure 2C). Only few apoptotic chondrocytes were detected in sham-operated mice whereas an increased number of randomly distributed apoptotic chondrocytes was noticeable in menisectomized mice. Chondrocyte aptosis was similar between WT and KO mice (Figure 2C).

### MNX IL-1β^−/−^ mice have similar knee joint calcification but increased subchondral bone osteoporosis compared with WT mice

**Figure 3:**
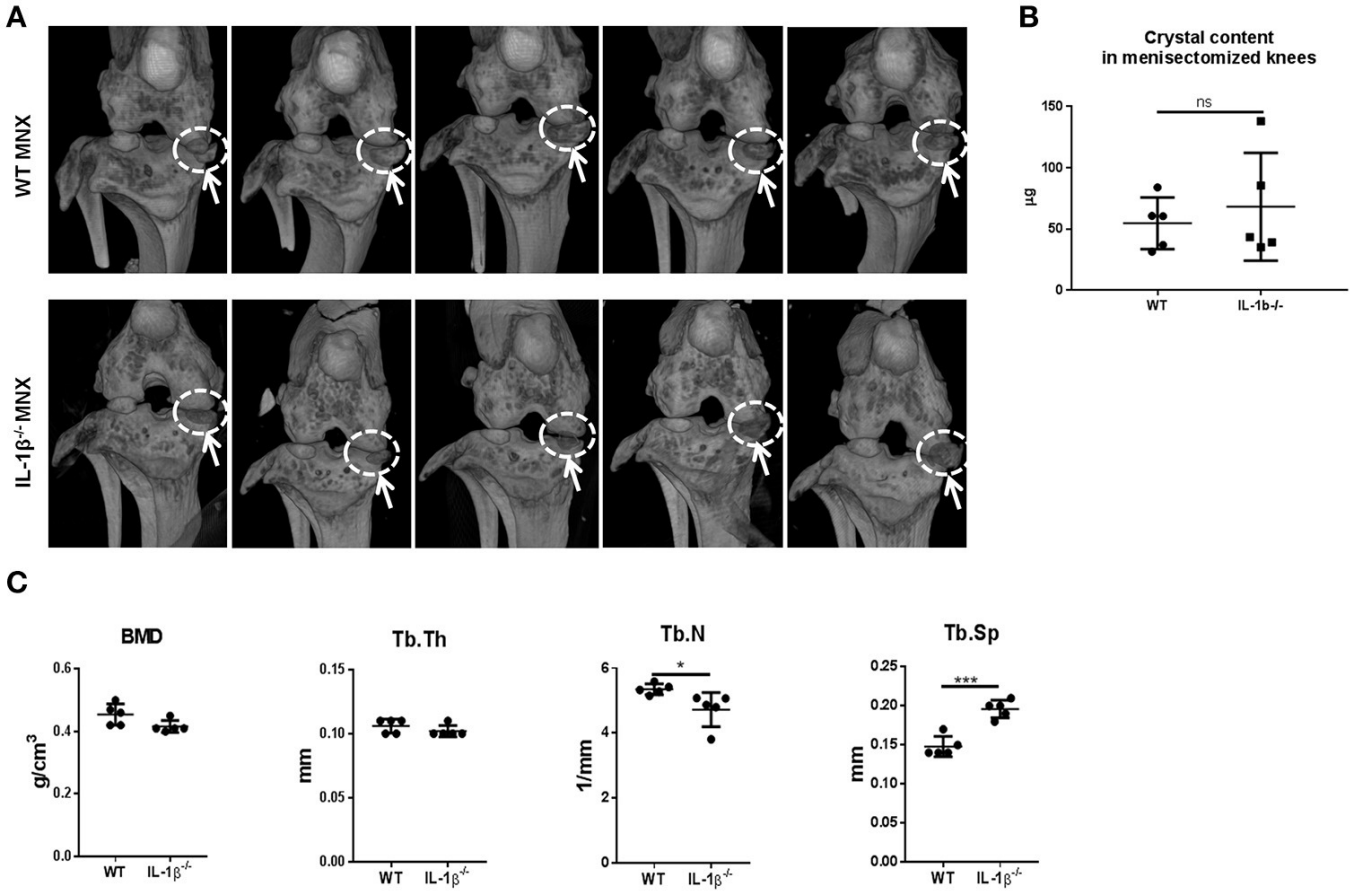
Calcific deposits and tibial subchondral trabecular bone parameters in osteoarthritic knee joints of WT and IL-1β^−/−^ mice. **(A)** Micro-CT scan images of menisectomized murine knee joints 2 months after surgery. White circles show new periarticular crystal deposits in menisectomized knees. **(B)** Crystal content in the menisectomized knees and **(C)** tibial subchondral trabecular bone parameters were measured in the same animals using CTAnalyzer. Mice number: WT *n* = 5, IL-1β^−/−^ *n* = 5. ^*^p < 0.05; ^***^p < 0.001.

We previously demonstrated that, 2 months after menisectomy, mice exhibited new calcific formation at the place of the removed meniscus (Nasi et al., 2016a). Calcification of the joint structures is a typical OA feature, also found in human OA, and IL-1 can be a trigger of joint calcification as discussed above. We therefore examined if menisectomy-induced calcific deposits were different between WT and IL-1β^−/−^ mice. MicroCT-scan examination conducted 8 weeks after surgery revealed similar joint calcification (Figure 3A, white circles) and crystal content (Figure 3B) between the two mice genotypes, suggesting that the lack of IL-1β did not protect against ectopic mineralization induced by joint instability. Moreover, analysis of subchondral trabecular bone parameters revealed that IL-1β^−/−^ mice have similar tibial bone mineral density (BMD) and trabecular thickness (Tb.Th) to WT mice. However, they showed significantly decreased trabecular number (Tb.N) and increased trabecular separation (Tb.Sp), suggesting a high subchondral bone remodeling with increased bone resorption (Chiba et al., 2012) (Figure 3C).

### IL-1β induces chondrocyte mineralization, but mineralization does not induce IL-1β secretion

**Figure 4:**
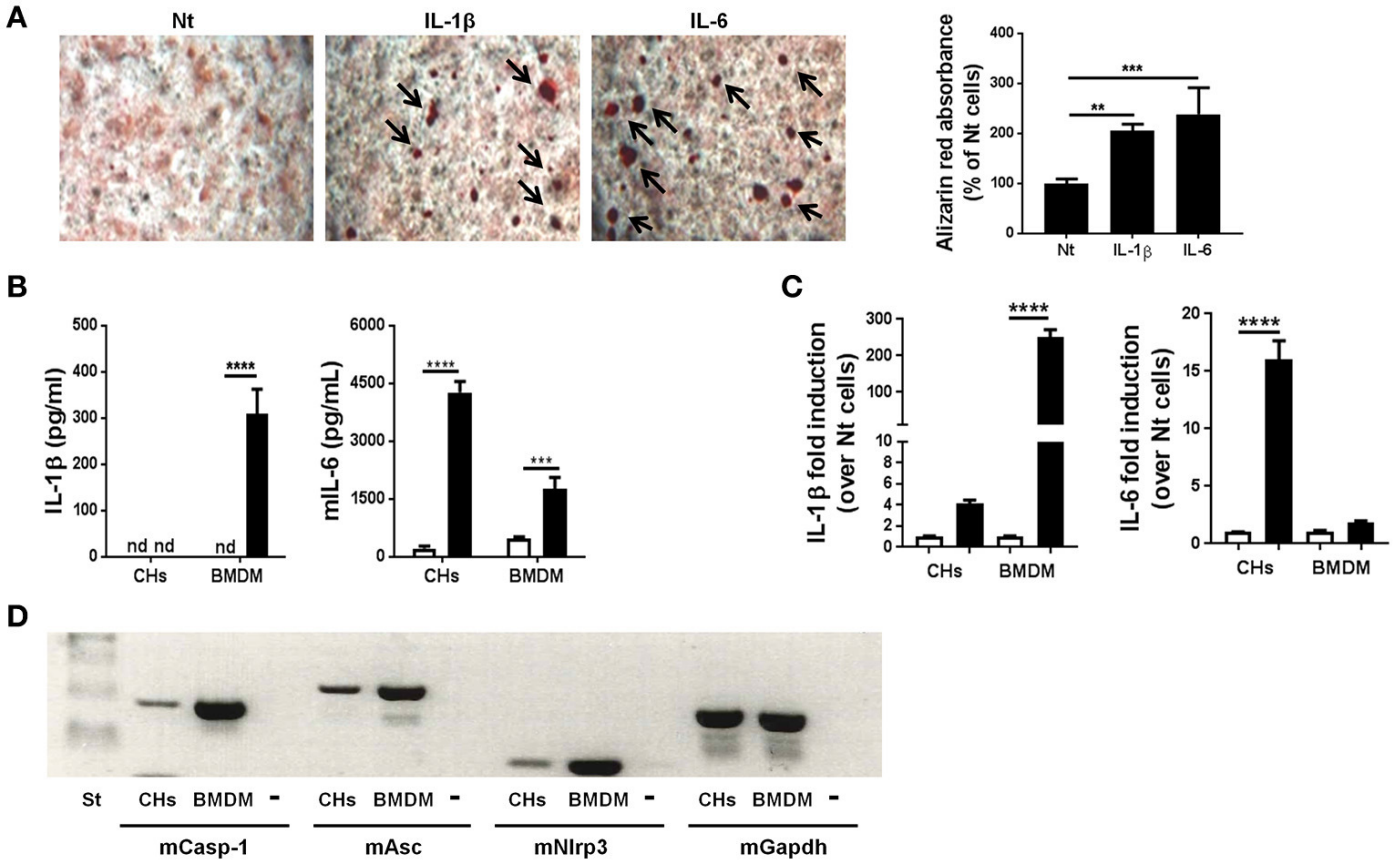
Murine primary chondrocytes express the components of the NLRP3 inflammasome but do not produce IL-1β upon HA crystal stimulation. **(A)** Alizarin red staining and quantification in murine chondrocytes culture, stimulated or not with 5 ng/ml IL-1β or with 10 ng/ml IL-6 for 7 days in BGJb medium. Values represent means ± *SD* of triplicates from one representative experiment out of three. **(B)** IL-1β and IL-6 secretion by primed CHs or by BMDM, stimulated (black bars) or not (white bars) with HA crystals for 6 h. Values represent means ± *SD* of triplicates from one representative experiment out of three. **(C)** qRT-PCR analysis of the indicated genes in CHs and BMDM stimulated (black bars) or not (white bars) with 100 μg/ml PAM3Cys for 2 h. Results are expressed as the fold increase of gene expression in PAM3Cys treated over not treated (Nt) cells, using the mean ± *SD* of triplicate independent RNA samples. **(D)** Gel electrophoresis for the analysis of Caspase-1, Asc and Nlrp3 expression in primary murine chondrocytes (CHs) and primary bone marrow derived macrophages (BMDM). Minus (-) is the negative control (water). All experiments were performed using pools of primary cells from at least 3 different mice. ^**^p < 0.01, ^***^p < 0.001, ^****^p < 0.0001.

We previously demonstrated that BCP crystals stimulated IL-6 secretion by murine chondrocytes. Conversely, exogenous IL-6 promoted chondrocyte mineralization, thus building an amplification loop leading to OA (Nasi et al., 2016a). We hypothesized that the absence of IL-1 effect in the MNX model could be due to the absence of this amplification loop. To test this hypothesis, we stimulated primary murine chondrocytes (CHs) with exogenous IL-1β. After 7 days of culture, calcium containing crystals, detected by Alizarin red staining were significantly increased compared to unstimulated cells (Figure 4A). As a positive control, we used IL-6 incubated for 7 days, which showed an even stronger promineralizing activity (Figure 4A). We next stimulated primed CHs and BMDM with HA crystals. In these conditions, chondrocytes did not secrete mature IL-1β, but an abundant secretion of IL-6 could be detected. By contrast, increased levels of IL-1β and IL-6 were measurable in HA-stimulated BMDM (Figure 4B). To explain the ELISA results, we analyzed the effect of the PAM3Cys priming on IL-1β and IL-6 genes. qRT-PCR analysis revealed that PAM3Cys strongly induced *IL-1*β gene expression in BMDM, but only marginally in CHs, whereas priming had an opposite effect on *IL-6* gene expression, being strongly up-modulated in CHs, but almost not in BMDM (Figure 4C). Therefore, the lack of IL-1β detection in primed CHs, under basal and HA-stimulated conditions, could be due to lower *IL-1*β gene expression in CHs (35<Ct<40) compared to BMDM (28<Ct<30). Additionally, the absence of IL-1β detection could also be accounted for by lower expression of NLRP3 inflammasome components in CHs compared to BMDM (Figure 4D). Our results are in agreement with already reported lower NLRP3 inflammasome expression in human OA chondrocytes and synoviocytes compared to the monocytic THP1 (Jin et al., 2011).

## Discussion

As *in vitro* IL-1 stimulation of joint cells led to inflammation, catabolism and oxidative stress, and IL-1 blockade by IL-1Ra or IL-1R2 reverted these IL-1-induced deleterious effects (Roessler et al., 1995; Attur et al., 2000, 2002; Palmer et al., 2002, 2004), it has been suggested for more than two decades that *in vivo* IL-1 could be of paramount significance in OA (Fernandes et al., 2002). In the present study, we demonstrated that IL-1β^−/−^ and NLRP3^−/−^ mice were not protected against cartilage damage and synovial inflammation induced by menisectomy. Rather, cartilage damage was exacerbated by these deficiencies, thereby confirming previous results obtained in a similar MNX model, in which cartilage was more damaged in IL-1β^−/−^ and caspase-1^−/−^ mice compared to WT mice (Clements et al., 2003). The mechanisms explaining the protective role of IL-1β and NLRP3 inflammasome on cartilage in the surgically induced model of OA remain to be explored. We also reported here that IL-1α is not involved in cartilage damage and synovial inflammation as, in the MNX model, the phenotype of IL-1α^−/−^ was similar to that of WT mice. In agreement with the lack of a protective role of IL-1 deficiency in experimental OA, in another model of experimental OA, the collagenase-induced model of OA, mice deficient for both IL-1α and IL-1β (IL-1αβ^−/−^) developed cartilage destruction and synovial inflammation similar to WT mice (van Dalen et al., 2016). Interestingly, histological scoring of the cartilage lesion showed a trend toward increased damage in IL-1 deficient mice, although this increase did not reach significancy. The lack of a pathogenic role of IL-1 has been further confirmed in the collagenase-induced model by the lack of effect of IL-1Ra treatment in WT osteoarthritic mice (van Dalen et al., 2016). We previously reported that intra-articular BCP crystals can elicit synovial inflammation and cartilage degradation suggesting that BCP crystals have a direct pathogenic role in OA. We also found that these effects are independent of IL-1 and NLRP3 inflammasome, as knee joint inflammation and damage was similar in crystal-injected IL-1α^−/−^, IL-1β^−/−^, ASC^−/−^, or NLRP3^−/−^ mice and as IL-1Ra treatment did not prevent OA features of WT mice (Ea et al., 2013). Finally, the fact that deficiency of MyD88, the adaptor molecule for IL-1R1, did not impact on the severity of experimental OA strongly suggests that IL-1 is not a key mediator in the development of OA (Nasi et al., 2014). Altogether our results strongly suggest that IL-1 is not involved in cartilage degeneration in murine models of OA. By contrast, in a spontaneous model of OA characterized by joint calcification (Ank^−/−^ model), NLRP3 inflammasome deficiency partially (~30%) protected against joint pathology (Jin et al., 2011). However, no proof of IL-1 involvement was provided in this study. Of note, one single report claimed that IL-1β^−/−^ mice had reduced cartilage erosion, but the number of mice and detailed procedures used to reach this conclusion were not mentioned and therefore caution should be taken when quoting this work (Glasson, 2007).

In non-murine experimental models of OA the role of IL1-mediated pathway seemed to be deleterious (see Table 2 for a summary of the works published). In the transection of the anterior cruciate ligament (ACL) dog model of OA, recombinant human interleukin-1-receptor antagonist (rHuIL-1Ra) either injected intra-articularly or locally expressed by synovial cells transduced with HuIL-1Ra gene protected against OA lesions, partially via a reduction of collagenase-1 expression (Caron et al., 1996; Pelletier et al., 1997). Similarly, in rabbit and equine surgically-induced models of OA, intra-articular overexpression of IL-1Ra resulted in a significant improvement in disease activity, cartilage degradation and synovitis (Fernandes et al., 1999; Frisbie et al., 2002). In a spontaneous model of OA (aged Hartley guinea pigs), diminished IL-1 signaling by RNA interference-based reduction (siIL-1β), or by mILRa led to decreases expression of mediators implicated in OA pathogenesis such as IL-1β, TNF-α, IL-8, INF-γ, MMP-13, and increased TGF-β1 (Santangelo et al., 2012). Finally, in a murine model of post-traumatic arthritis induced by articular fracture, intra-articular inhibition of IL-1 by IL-1Ra exerted protective effects in terms of cartilage degradation and synovial inflammation (Furman et al., 2014).

**Table 2:** Summary of studies in animal models of OA where IL-1 role has been tested.

**Species**	**Model**	**Conditions**	**Duration of experiment**	**Conclusion about IL-1 role in OA**	**Citation**
Dog	ACL transection	I.a injection of 4 mg rHuIL-1Ra, at the moment of surgery	4 weeks	Deleterious	Caron et al., 1996
Dog	ACL transection	I.a injection of autologous synoviocytes transduced with HuIL-1Ra, 2d post-surgery	4 weeks	Deleterious	Pelletier et al., 1997
Rabbit	Partial medial menisectomy	Three i.a injection at 24 h intervals of 1,000 μg DogIL-Ra plasmid, 4w post-surgery	8 weeks	Deleterious	Fernandes et al., 1999
Horse	Osteochondral fragment	I.a injection of 20 × 10^10^ VP AdEqIL-1Ra, 2w post-surgery	10 weeks	Deleterious	Frisbie et al., 2002
Guinea pig	Spontaneous	I.a injection of 10^12^ DRPs siIL-1β, at 8w of age	24 weeks	Deleterious	Santangelo et al., 2012
		I.a injection of 2x10^11^ IFUs mAd-IL-1Ra, at 8w of age	24 weeks	Deleterious	
Mouse	Destabilization medial meniscus	IL-1β^−/−^ mice	8 weeks	Deleterious	Glasson, 2007
Mouse	Ank^−/−^	Caspase-1^−/−^ mice	12 weeks	Deleterious	Jin et al., 2011
		NLRP3^−/−^ mice			
Mouse	Articular fracture of the knee	IL-1Ra administration intra-articularly or sistemically	4 weeks	Deleterious	Furman et al., 2014
Mouse	I.a injection of BCP crystals	IL-1α^−/−^, IL-1β^−/−^, ASC^−/−^, NLRP3^−/−^ mice	4, 17, 30 days	No role No role	Ea et al., 2013
		IL-1Ra	4 days		
Mouse	Partial medial menisectomy	IL-1β^−/−^ mice	4 weeks	Protective	Clements et al., 2003
		Caspase-1^−/−^ mice	4 weeks	Protective	
Mouse	Partial medial menisectomy	MyD88^−/−^ mice	8 weeks	No role	Nasi et al., 2014
Mouse	Partial medial menisectomy	IL-1α^−/−^ mice	8 weeks	No role	This study
		IL-1β^−/−^ mice	8 weeks	Protective	
		NLRP3^−/−^ mice	8 weeks	Protective	
Mouse	Collagenase-induced	IL-1αβ^−/−^ mice	4 weeks	No role	van Dalen et al., 2016
		rIL-1Ra administration at the moment of the surgery and for 2w	2 weeks		

The conflicting results about the role of IL-1, but also other cytokines such as IL-6 (de Hooge et al., 2005; Ryu et al., 2011; Latourte et al., 2016; Nasi et al., 2016a) in experimental models of OA, could reflect species difference, differences in age and sex of the animals used, variability in the method of OA induction (different progression of joint degeneration, degrees of inflammation, degrees of unpaired loading for each experimental OA model).”

It is important to keep in mind that in the context of inflammatory arthritis, such as collagen-induced arthritis (Joosten et al., 1996) and antigen-induced arthritis (Kolly et al., 2009), IL-1 deficiency or IL-1 neutralization leads to cartilage protection. This suggests that the mechanisms involved in cartilage degradation in an inflammatory context may be dependent on IL-1 whereas in a less inflammatory setting (such as in OA) this could not be any more true.

Discordant results about the role of IL-1 in OA have been reported not only in animal studies but also in human studies. It has been reported that patients subjected to ACL transection have increased IL-1β and IL-1Ra in synovial fluid, if compared to healthy individuals, while IL-1α remained undetectable (Marks and Donaldson, 2005). In accordance, the expression of IL-1β in synovial membrane positively correlated with OA grade (Smith et al., 1997) and with joint space width (Ning et al., 2011), and negatively correlated with joint alignment and physical disability (Ning et al., 2011). Moreover, IL-1α and IL-1β expression was detected by immunohistochemical analysis in human OA cartilage, especially in early stage OA (Towle et al., 1997). In a study by Denoble and co-workers, IL-1β level in synovial fluid of OA patients was increased and correlated with synovial fluid uric acid. The author concluded that uric acid could be a danger signal that contributes to increasing risk for OA through inflammasome activation and subsequent IL-1β production (Denoble et al., 2011). On the contrary, in a study conducted at different time-points after ACL injury, synovial fluid level of IL-1β was not increased and IL-1Ra was decreased compared to healthy controls (Bigoni et al., 2013). In a study in symptomatic knee OA patients, plasma levels of IL-1Ra were modestly associated with the severity and progression of the disease independent of other risk factors (Attur et al., 2015). High innate *ex vivo* production of IL-1β and IL-1Ra by whole blood samples from OA patients was associated with an increased risk of familial OA at multiple sites (Riyazi et al., 2005). However, in a separate study, *ex vivo* production of IL-1β and IL-1Ra by whole blood samples were not significantly associated with progression of knee OA over a 2-year period (Botha-Scheepers et al., 2008).

In addition to the contrasting results about the role of IL-1 in OA obtained in a number of experimental studies *in vitro, in vivo*, and *ex vivo*, the anti-IL-1 approach in patients has not yet proven significative improvement in OA symptoms and as a disease-modifying therapy. Various treatment strategies have been tested in human such as administration of monoclonal antibodies against IL-1 or IL-1R1 to block IL-1 signaling, administration of IL-1Ra to antagonize IL-1 action, and blockade of the formation of active IL-1β. In a first double-blind, placebo controlled, multiple-dose study by Cohen and colleagues (Cohen et al., 2011), a monoclonal antibody (AMG108) directed against IL-1R1, therefore inhibiting both IL-1α and IL-1β activity, was administered systemically (IV or SC) to knee OA patients (KOA). AMG108-treated group did not show statistically significant improvements in pain compared with the placebo group, as shown by pain scores (Cohen et al., 2011). Recombinant human IL-1 receptor antagonist proteins (IRAPs) are competitive antagonists of IL-1. In placebo-controlled clinical trials conducted in KOA patients, intra-articular injection of either Anakinra or Orthokine, two available IRAPs, didn't lead to significantly different pain score compared to placebo-treated patients (Chevalier et al., 2005, 2009; Auw Yang et al., 2008). In another study in KOA patients, the clinical effects of intra-articular injection of Orthokine was compared with those of hyaluronan (HA) or placedo injection (Baltzer et al., 2009). Preliminary results showed that the effects of Orthokine were higher than those of HA or saline in terms of pain, stiffness and joint function (about 30% improvement) (Baltzer et al., 2009). Further confirmation and additional studies on the mechanism of action of Orthokine (i.e., DMOAD, chondroprotective, others) are required.

**Figure 5:**
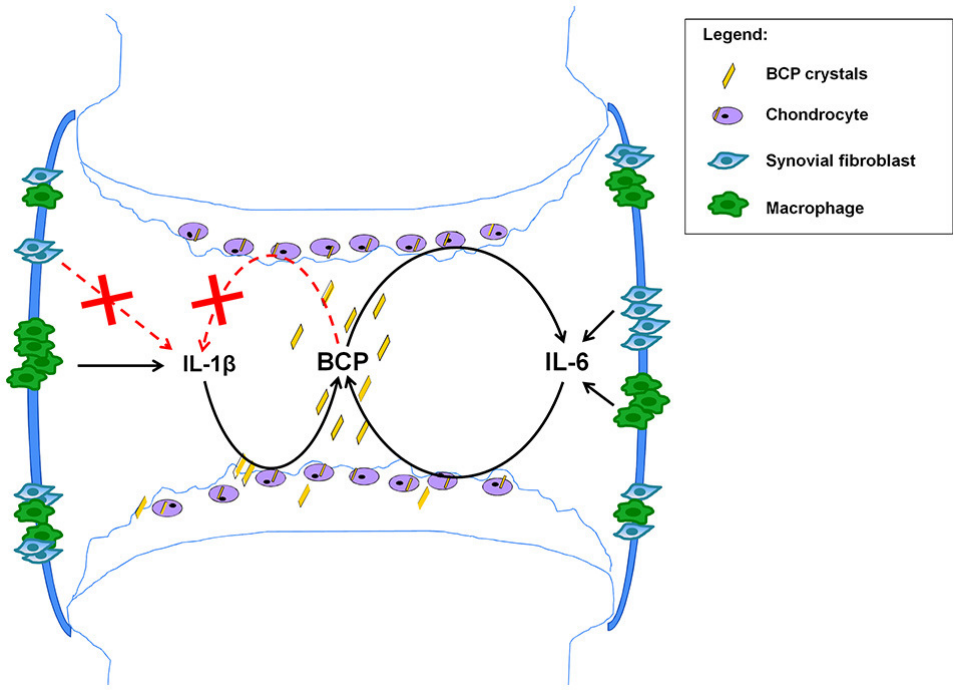
Proposed mechanism based on the obtained results. BCP crystals found in 100% of OA patients at the moment of joint replacement (Ea et al., 2011), can activate NLRP3 inflammasome in macrophages leading to IL-1β secretion (Pazar et al., 2011), but this process does not occur in chondrocytes and synovial fibroblasts (Kolly et al., 2010; Jin et al., 2011). In addition BCP crystals induce IL-6 in macrophages, fibroblasts and chondrocytes. In these latter cells, BCP crystals induce IL-6, which in turn induce mineralization, thus creating a vicious circle and a chronification of the disease. Strategies interrupting this vicious circle could ameliorate this degenerative disease.

From the above mentioned data obtained both in experimental and clinical OA, we can conclude that IL-1 has not yet proven to be a good target for OA. Based on previous published papers by us (Nasi et al., 2016a,b) and by others (Ryu et al., 2011; Latourte et al., 2016), we suggest that IL-6-targeted strategies could lead to new therapeutic options for OA, by interrupting the vicious circle between BCP crystal formation and IL-6 production by chondrocytes (see Figure 5).

This proposed mechanism, built on chondrocytes grown in monolayers and *in vivo* observations in the menisectomy model after 2 months, needs further validations using additional *in vitro* models, such 3D-models of chondrocyte cultures, and additional *in vivo* OA models, considering different experimental points and looking to different read-outs.

## Ethics statement

The experiments complied with the Guidelines for Animal Experimentation issued by the local Ethics Committee on Animal Care and Experimentation.

## Author contributions

SN, HE, and NB designed, performed, and evaluated all experiments. The entire work was supervised by NB. The figures were prepared and the manuscript was written by SN, HE, AS, and NB. All authors discussed and commented on the manuscript.

### Conflict of interest statement

The authors declare that the research was conducted in the absence of any commercial or financial relationships that could be construed as a potential conflict of interest.

## Abbreviations

MyD88: Myeloid differentiation primary response gene 88
IL-1α: Interleukin-1α
IL-1β: Interleukin-1β
NLRP3: NOD-like receptor protein-3
OA: Osteoarthritis
MNX: Medial partial menisectomy.

## References

[B1] Abramson S. B. Attur M. (2009). Developments in the scientific understanding of osteoarthritis. Arthritis Res. Ther. 11, 227. 10.1186/ar2655 19519925 PMC2714096

[B2] Attur M. G. Dave M. N. Leung M. Y. Cipolletta C. Meseck M. Woo S. L.. (2002). Functional genomic analysis of type II IL-1beta decoy receptor: potential for gene therapy in human arthritis and inflammation. J. Immunol. 168, 2001–2010. 10.4049/jimmunol.168.4.2001 11823537

[B3] Attur M. G. Dave M. Cipolletta C. Kang P. Goldring M. B. Patel I. R.. (2000). Reversal of autocrine and paracrine effects of interleukin 1 (IL-1) in human arthritis by type II IL-1 decoy receptor. Potential for pharmacological intervention. J. Biol. Chem. 275, 40307–40315. 10.1074/jbc.M002721200 11007768

[B4] Attur M. Statnikov A. Samuels J. Li Z. Alekseyenko A. V. Greenberg J. D.. (2015). Plasma levels of interleukin-1 receptor antagonist (IL1Ra) predict radiographic progression of symptomatic knee osteoarthritis. Osteoarthritis Cartilage 23, 1915–1924. 10.1016/j.joca.2015.08.006 26521737 PMC4630783

[B5] Auw Yang K. G. Raijmakers N. J. van Arkel E. R. Caron J. J. Rijk P. C. Willems W. J.. (2008). Autologous interleukin-1 receptor antagonist improves function and symptoms in osteoarthritis when compared to placebo in a prospective randomized controlled trial. Osteoarthritis Cartilage 16, 498–505. 10.1016/j.joca.2007.07.008 17825587

[B6] Baltzer A. W. Moser C. Jansen S. A. Krauspe R. (2009). Autologous conditioned serum (Orthokine) is an effective treatment for knee osteoarthritis. Osteoarthritis Cartilage 17, 152–160. 10.1016/j.joca.2008.06.014 18674932

[B7] Bigoni M. Sacerdote P. Turati M. Franchi S. Gandolla M. Gaddi D.. (2013). Acute and late changes in intraarticular cytokine levels following anterior cruciate ligament injury. J. Orthop. Res. 31, 315–321. 10.1002/jor.22208 22886741

[B8] Botha-Scheepers S. Watt I. Slagboom E. de Craen A. J. Meulenbelt I. Rosendaal F. R.. (2008). Innate production of tumour necrosis factor α and interleukin 10 is associated with radiological progression of knee osteoarthritis. Ann. Rheum. Dis. 67, 1165–1169. 10.1136/ard.2007.084657 18029383

[B9] Broz P. Dixit V. M. (2016). Inflammasomes: mechanism of assembly, regulation and signalling. Nat. Rev. Immunol. 16, 407–420. 10.1038/nri.2016.58 27291964

[B10] Caron J. P. Fernandes J. C. Martel-Pelletier J. Tardif G. Mineau F. Geng C.. (1996). Chondroprotective effect of intraarticular injections of interleukin-1 receptor antagonist in experimental osteoarthritis. Suppression of collagenase-1 expression. Arthritis Rheum. 39, 1535–1544. 10.1002/art.1780390914 8814066

[B11] Chevalier X. Giraudeau B. Conrozier T. Marliere J. Kiefer P. Goupille P. (2005). Safety study of intraarticular injection of interleukin 1 receptor antagonist in patients with painful knee osteoarthritis: a multicenter study. J. Rheumatol. 32, 1317–1323. 15996071

[B12] Chevalier X. Goupille P. Beaulieu A. D. Burch F. X. Bensen W. G. Conrozier T.. (2009). Intraarticular injection of anakinra in osteoarthritis of the knee: a multicenter, randomized, double-blind, placebo-controlled study. Arthritis Rheum. 61, 344–352. 10.1002/art.24096 19248129

[B13] Chiba K. Uetani M. Kido Y. Ito M. Okazaki N. Taguchi K.. (2012). Osteoporotic changes of subchondral trabecular bone in osteoarthritis of the knee: a 3-T MRI study. Osteoporosis Int. 23, 589–597. 10.1007/s00198-011-1585-2 21359670

[B14] Clements K. M. Price J. S. Chambers M. G. Visco D. M. Poole A. R. Mason R. M. (2003). Gene deletion of either interleukin-1beta, interleukin-1beta-converting enzyme, inducible nitric oxide synthase, or stromelysin 1 accelerates the development of knee osteoarthritis in mice after surgical transection of the medial collateral ligament and partial medial meniscectomy. Arthritis Rheum. 48, 3452–3463. 10.1002/art.11355 14673996

[B15] Cohen S. B. Proudman S. Kivitz A. J. Burch F. X. Donohue J. P. Burstein D.. (2011). A randomized, double-blind study of AMG 108 (a fully human monoclonal antibody to IL-1R1) in patients with osteoarthritis of the knee. Arthritis Res. Ther. 13, R125. 10.1186/ar3430 21801403 PMC3239365

[B16] de Hooge A. S. van de Loo F. A. Bennink M. B. Arntz O. J. de Hooge P. van den Berg W. B. (2005). Male IL-6 gene knock out mice developed more advanced osteoarthritis upon aging. Osteoarthritis Cartilage 13, 66–73. 10.1016/j.joca.2004.09.011 15639639

[B17] Denoble A. E. Huffman K. M. Stabler T. V. Kelly S. J. Hershfield M. S. McDaniel G. E.. (2011). Uric acid is a danger signal of increasing risk for osteoarthritis through inflammasome activation. Proc. Natl. Acad. Sci. U.S.A. 108, 2088–2093. 10.1073/pnas.1012743108 21245324 PMC3033282

[B18] Di Paolo N. C. Shayakhmetov D. M. (2016). Interleukin 1α and the inflammatory process. Nat. Immunol. 17, 906–913. 10.1038/ni.3503 27434011 PMC5152572

[B19] Ea H. K. Liote F. (2009). Advances in understanding calcium-containing crystal disease. Curr. Opin. Rheumatol. 21, 150–157. 10.1097/BOR.0b013e3283257ba9 19339926

[B20] Ea H. K. Chobaz V. Nguyen C. Nasi S. van Lent P. Daudon M.. (2013). Pathogenic role of basic calcium phosphate crystals in destructive arthropathies. PLoS ONE 8:e57352. 10.1371/journal.pone.0057352 23468973 PMC3585350

[B21] Ea H. K. Nguyen C. Bazin D. Bianchi A. Guicheux J. Reboul P.. (2011). Articular cartilage calcification in osteoarthritis: insights into crystal-induced stress. Arthritis Rheum. 63, 10–18. 10.1002/art.27761 20862682

[B22] Fernandes J. C. Martel-Pelletier J. Pelletier J. P. (2002). The role of cytokines in osteoarthritis pathophysiology. Biorheology 39, 237–246. 12082286

[B23] Fernandes J. Tardif G. Martel-Pelletier J. Lascau-Coman V. Dupuis M. Moldovan F.. (1999). *In vivo* transfer of interleukin-1 receptor antagonist gene in osteoarthritic rabbit knee joints: prevention of osteoarthritis progression. Am. J. Pathol. 154, 1159–1169. 10.1016/S0002-9440(10)65368-0 10233854 PMC1866546

[B24] Frisbie D. D. Ghivizzani S. C. Robbins P. D. Evans C. H. McIlwraith C. W. (2002). Treatment of experimental equine osteoarthritis by *in vivo* delivery of the equine interleukin-1 receptor antagonist gene. Gene Ther. 9, 12–20. 10.1038/sj.gt.3301608 11850718

[B25] Furman B. D. Mangiapani D. S. Zeitler E. Bailey K. N. Horne P. H. Huebner J. L.. (2014). Targeting pro-inflammatory cytokines following joint injury: acute intra-articular inhibition of interleukin-1 following knee injury prevents post-traumatic arthritis. Arthritis Res. Ther. 16, R134. 10.1186/ar4591 24964765 PMC4229982

[B26] Gibilisco P. A. Schumacher H. R. Jr. Hollander J. L. Soper K. A. (1985). Synovial fluid crystals in osteoarthritis. Arthritis Rheum. 28, 511–515. 10.1002/art.1780280507 2988572

[B27] Glasson S. S. (2007). *In vivo* osteoarthritis target validation utilizing genetically-modified mice. Curr. Drug Targets 8, 367–376. 10.2174/138945007779940061 17305514

[B28] Glasson S. S. Chambers M. G. Van Den Berg W. B. Little C. B. (2010). The OARSI histopathology initiative - recommendations for histological assessments of osteoarthritis in the mouse. Osteoarthritis Cartilage 18(Suppl. 3), S17–S23. 10.1016/j.joca.2010.05.025 20864019

[B29] Goldring M. B. (2006). Update on the biology of the chondrocyte and new approaches to treating cartilage diseases. Best Pract. Res. Clin. Rheumatol. 20, 1003–1025. 10.1016/j.berh.2006.06.003 16980220

[B30] Goldring M. B. Goldring S. R. (2007). Osteoarthritis. J. Cell. Physiol. 213, 626–634. 10.1002/jcp.21258 17786965

[B31] Goldring M. B. Birkhead J. Sandell L. J. Kimura T. Krane S. M. (1988). Interleukin 1 suppresses expression of cartilage-specific types II and IX collagens and increases types I and III collagens in human chondrocytes. J. Clin. Invest. 82, 2026–2037. 10.1172/JCI113823 3264290 PMC442785

[B32] Gosset M. Berenbaum F. Thirion S. Jacques C. (2008). Primary culture and phenotyping of murine chondrocytes. Nat. Protoc. 3, 1253–1260. 10.1038/nprot.2008.95 18714293

[B33] Gregory C. A. Gunn W. G. Peister A. Prockop D. J. (2004). An Alizarin red-based assay of mineralization by adherent cells in culture: comparison with cetylpyridinium chloride extraction. Anal. Biochem. 329, 77–84. 10.1016/j.ab.2004.02.002 15136169

[B34] Gross O. Yazdi A. S. Thomas C. J. Masin M. Heinz L. X. Guarda G.. (2012). Inflammasome activators induce interleukin-1α secretion via distinct pathways with differential requirement for the protease function of caspase-1. Immunity 36, 388–400. 10.1016/j.immuni.2012.01.018 22444631

[B35] Hashizume M. Mihara M. (2010). High molecular weight hyaluronic acid inhibits IL-6-induced MMP production from human chondrocytes by up-regulating the ERK inhibitor, MKP-1. Biochem. Biophys. Res. Commun. 403, 184–189. 10.1016/j.bbrc.2010.10.135 21059338

[B36] Iqbal I. Fleischmann R. (2007). Treatment of osteoarthritis with anakinra. Curr. Rheumatol. Rep. 9, 31–35. 10.1007/s11926-007-0019-9 17437664

[B37] Jandinski J. J. (1988). Osteoclast activating factor is now interleukin-1 beta: historical perspective and biological implications. J. Oral Pathol. 17, 145–152. 10.1111/j.1600-0714.1988.tb01515.x 3139850

[B38] Jin C. Frayssinet P. Pelker R. Cwirka D. Hu B. Vignery A.. (2011). NLRP3 inflammasome plays a critical role in the pathogenesis of hydroxyapatite-associated arthropathy. Proc. Natl. Acad. Sci. U.S.A. 108, 14867–14872. 10.1073/pnas.1111101108 21856950 PMC3169126

[B39] Johnson K. Hashimoto S. Lotz M. Pritzker K. Terkeltaub R. (2001). Interleukin-1 induces pro-mineralizing activity of cartilage tissue transglutaminase and factor XIIIa. Am. J. Pathol. 159, 149–163. 10.1016/S0002-9440(10)61682-3 11438463 PMC1850418

[B40] Joosten L. A. Helsen M. M. van de Loo F. A. van den Berg W. B. (1996). Anticytokine treatment of established type II collagen-induced arthritis in DBA/1 mice. A comparative study using anti-TNF alpha, anti-IL-1 alpha/beta, and IL-1Ra. Arthritis Rheum. 39, 797–809. 8639177 10.1002/art.1780390513

[B41] Jules J. Zhang P. Ashley J. W. Wei S. Shi Z. Liu J.. (2012). Molecular basis of requirement of receptor activator of nuclear factor kappaB signaling for interleukin 1-mediated osteoclastogenesis. J. Biol. Chem. 287, 15728–15738. 10.1074/jbc.M111.296228 22416138 PMC3346127

[B42] Kim J. H. Jin H. M. Kim K. Song I. Youn B. U. Matsuo K.. (2009). The mechanism of osteoclast differentiation induced by IL-1. J. Immunol. 183, 1862–1870. 10.4049/jimmunol.0803007 19587010

[B43] Kolly L. Busso N. Palmer G. Talabot-Ayer D. Chobaz V. So A. (2010). Expression and function of the NALP3 inflammasome in rheumatoid synovium. Immunology 129, 178–185. 10.1111/j.1365-2567.2009.03174.x 19824913 PMC2814460

[B44] Kolly L. Karababa M. Joosten L. A. Narayan S. Salvi R. Petrilli V.. (2009). Inflammatory role of ASC in antigen-induced arthritis is independent of caspase-1, NALP-3, and IPAF. J. Immunol. 183, 4003–4012. 10.4049/jimmunol.0802173 19717512

[B45] Latourte A. Cherifi C. Maillet J. Ea H. K. Bouaziz W. Funck-Brentano T.. (2016). Systemic inhibition of IL-6/Stat3 signalling protects against experimental osteoarthritis. Ann. Rheum. Dis. 76, 1–8. 10.1136/annrheumdis-2016-209757 27789465

[B46] Livshits G. Zhai G. Hart D. J. Kato B. S. Wang H. Williams F. M.. (2009). Interleukin-6 is a significant predictor of radiographic knee osteoarthritis: the Chingford Study. Arthritis Rheum. 60, 2037–2045. 10.1002/art.24598 19565477 PMC2841820

[B47] Loeser R. F. Goldring S. R. Scanzello C. R. Goldring M. B. (2012). Osteoarthritis: a disease of the joint as an organ. Arthritis Rheum. 64, 1697–1707. 10.1002/art.34453 22392533 PMC3366018

[B48] Marks P. H. Donaldson M. L. (2005). Inflammatory cytokine profiles associated with chondral damage in the anterior cruciate ligament-deficient knee. Arthroscopy 21, 1342–1347. 10.1016/j.arthro.2005.08.034 16325085

[B49] McCarthy G. M. Cheung H. S. (2009). Point: hydroxyapatite crystal deposition is intimately involved in the pathogenesis and progression of human osteoarthritis. Curr. Rheumatol. Rep. 11, 141–147. 10.1007/s11926-009-0020-6 19296887

[B50] McNulty A. L. Rothfusz N. E. Leddy H. A. Guilak F. (2013). Synovial fluid concentrations and relative potency of interleukin-1 alpha and beta in cartilage and meniscus degradation. J. Orthop. Res. 31, 1039–1045. 10.1002/jor.22334 23483596 PMC4037157

[B51] Murphy G. Nagase H. (2008). Reappraising metalloproteinases in rheumatoid arthritis and osteoarthritis: destruction or repair? Nat. Clin. Pract. Rheumatol. 4, 128–135. 10.1038/ncprheum0727 18253109

[B52] Nakamura I. Jimi E. (2006). Regulation of osteoclast differentiation and function by interleukin-1. Vitam. Horm. 74, 357–370. 10.1016/S0083-6729(06)74015-8 17027523

[B53] Nasi S. Ea H. K. Chobaz V. van Lent P. Liote F. So A.. (2014). Dispensable role of myeloid differentiation primary response gene 88 (MyD88) and MyD88-dependent toll-like receptors (TLRs) in a murine model of osteoarthritis. Joint Bone Spine 81, 320–324. 10.1016/j.jbspin.2014.01.018 24703622

[B54] Nasi S. Ea H. K. Liote F. So A. Busso N. (2016b). Sodium thiosulfate prevents chondrocyte mineralization and reduces the severity of murine osteoarthritis. PLoS ONE 11:e0158196. 10.1371/journal.pone.0158196 27391970 PMC4938519

[B55] Nasi S. So A. Combes C. Daudon M. Busso N. (2016a). Interleukin-6 and chondrocyte mineralisation act in tandem to promote experimental osteoarthritis. Ann. Rheum. Dis. 75, 1372–1379. 10.1136/annrheumdis-2015-207487 26253096

[B56] Ning L. Ishijima M. Kaneko H. Kurihara H. Arikawa-Hirasawa E. Kubota M.. (2011). Correlations between both the expression levels of inflammatory mediators and growth factor in medial perimeniscal synovial tissue and the severity of medial knee osteoarthritis. Int. Orthop. 35, 831–838. 10.1007/s00264-010-1045-1 20517696 PMC3103960

[B57] Palmer G. Guerne P. A. Mezin F. Maret M. Guicheux J. Goldring M. B. (2002). Production of interleukin-1 receptor antagonist by human articular chondrocytes. Arthritis Res. 4, 226–231. 10.1186/ar411 12010575 PMC111027

[B58] Palmer G. Mezin F. Juge-Aubry C. E. Plater-Zyberk C. Gabay C. Guerne P. A. (2004). Interferon beta stimulates interleukin 1 receptor antagonist production in human articular chondrocytes and synovial fibroblasts. Ann. Rheum. Dis. 63, 43–49. 10.1136/ard.2002.005546 14672890 PMC1754734

[B59] Pazar B. Ea H. K. Narayan S. Kolly L. Bagnoud N. Chobaz V.. (2011). Basic calcium phosphate crystals induce monocyte/macrophage IL-1beta secretion through the NLRP3 inflammasome *in vitro*. J. Immunol. 186, 2495–2502. 10.4049/jimmunol.1001284 21239716

[B60] Pelletier J. P. Caron J. P. Evans C. Robbins P. D. Georgescu H. I. Jovanovic D.. (1997). *In vivo* suppression of early experimental osteoarthritis by interleukin-1 receptor antagonist using gene therapy. Arthritis Rheum. 40, 1012–1019. 10.1002/art.1780400604 9182910

[B61] Poree B. Kypriotou M. Chadjichristos C. Beauchef G. Renard E. Legendre F.. (2008). Interleukin-6 (IL-6) and/or soluble IL-6 receptor down-regulation of human type II collagen gene expression in articular chondrocytes requires a decrease of Sp1.Sp3 ratio and of the binding activity of both factors to the COL2A1 promoter. J. Biol. Chem. 283, 4850–4865. 10.1074/jbc.M706387200 18065760

[B62] Prudhommeaux F. Schiltz C. Liote F. Hina A. Champy R. Bucki B.. (1996). Variation in the inflammatory properties of basic calcium phosphate crystals according to crystal type. Arthritis Rheum. 39, 1319–1326. 10.1002/art.1780390809 8702440

[B63] Re F. Sironi M. Muzio M. Matteucci C. Introna M. Orlando S.. (1996). Inhibition of interleukin-1 responsiveness by type II receptor gene transfer: a surface “receptor” with anti-interleukin-1 function. J. Exp. Med. 183, 1841–1850. 10.1084/jem.183.4.1841 8666940 PMC2192538

[B64] Riyazi N. Slagboom E. de Craen A. J. Meulenbelt I. Houwing-Duistermaat J. J. Kroon H. M.. (2005). Association of the risk of osteoarthritis with high innate production of interleukin-1beta and low innate production of interleukin-10 *ex vivo*, upon lipopolysaccharide stimulation. Arthritis Rheum. 52, 1443–1450. 10.1002/art.21014 15880595

[B65] Roessler B. J. Hartman J. W. Vallance D. K. Latta J. M. Janich S. L. Davidson B. L. (1995). Inhibition of interleukin-1-induced effects in synoviocytes transduced with the human IL-1 receptor antagonist cDNA using an adenoviral vector. Hum. Gene Ther. 6, 307–316. 10.1089/hum.1995.6.3-307 7779914

[B66] Ryu J. H. Yang S. Shin Y. Rhee J. Chun C. H. Chun J. S. (2011). Interleukin-6 plays an essential role in hypoxia-inducible factor 2α-induced experimental osteoarthritic cartilage destruction in mice. Arthritis Rheum. 63, 2732–2743. 10.1002/art.30451 21590680

[B67] Santangelo K. S. Nuovo G. J. Bertone A. L. (2012). *In vivo* reduction or blockade of interleukin-1beta in primary osteoarthritis influences expression of mediators implicated in pathogenesis. Osteoarthritis Cartilage 20, 1610–1618. 10.1016/j.joca.2012.08.011 22935786 PMC3478416

[B68] Scanzello C. R. Goldring S. R. (2012). The role of synovitis in osteoarthritis pathogenesis. Bone 51, 249–257. 10.1016/j.bone.2012.02.012 22387238 PMC3372675

[B69] Smith M. D. Triantafillou S. Parker A. Youssef P. P. Coleman M. (1997). Synovial membrane inflammation and cytokine production in patients with early osteoarthritis. J. Rheumatol. 24, 365–371. 9034998

[B70] Sui Y. Lee J. H. DiMicco M. A. Vanderploeg E. J. Blake S. M. Hung H. H.. (2009). Mechanical injury potentiates proteoglycan catabolism induced by interleukin-6 with soluble interleukin-6 receptor and tumor necrosis factor α in immature bovine and adult human articular cartilage. Arthritis Rheum. 60, 2985–2996. 10.1002/art.24857 19790045

[B71] Towle C. A. Hung H. H. Bonassar L. J. Treadwell B. V. Mangham D. C. (1997). Detection of interleukin-1 in the cartilage of patients with osteoarthritis: a possible autocrine/paracrine role in pathogenesis. Osteoarthritis Cartilage 5, 293–300. 10.1016/S1063-4584(97)80008-8 9497936

[B72] van Beuningen H. M. Arntz O. J. van den Berg W. B. (1991). *In vivo* effects of interleukin-1 on articular cartilage. Prolongation of proteoglycan metabolic disturbances in old mice. Arthritis Rheum. 34, 606–615. 2025312 10.1002/art.1780340513

[B73] van Dalen S. C. Blom A. B. Sloetjes A. W. Helsen M. M. Roth J. Vogl T. (2016). Interleukin-1 is not involved in synovial inflammation and cartilage destruction in collagenase-induced osteoarthritis. Osteoarthritis Cartilage 25, 385–396. 10.1016/j.joca.2016.09.009 27654963

[B74] Wojdasiewicz P. Poniatowski L. A. Szukiewicz D. (2014). The role of inflammatory and anti-inflammatory cytokines in the pathogenesis of osteoarthritis. Mediators Inflamm. 2014:561459. 10.1155/2014/561459 24876674 PMC4021678

